# Investigating the Mechanical Characteristics of Bone-Metal Implant Interface Using *in situ* Synchrotron Tomographic Imaging

**DOI:** 10.3389/fbioe.2018.00208

**Published:** 2019-01-21

**Authors:** Sophie Le Cann, Erika Tudisco, Mikael J. Turunen, Alessandra Patera, Rajmund Mokso, Magnus Tägil, Ola Belfrage, Stephen A. Hall, Hanna Isaksson

**Affiliations:** ^1^Department of Biomedical Engineering, Lund University, Lund, Sweden; ^2^Division of Geotechnical Engineering, Lund University, Lund, Sweden; ^3^Department of Applied Physics, University of Eastern Finland, Kuopio, Finland; ^4^Swiss Light Source, Paul Scherrer Institute, Villigen, Switzerland; ^5^Max IV laboratory, Lund University, Lund, Sweden; ^6^Department of Orthopaedics, Clinical Sciences, Lund University, Lund, Sweden; ^7^Division of Solid Mechanics, Lund University, Lund, Sweden

**Keywords:** X-ray tomography, bone, metallic screw, *in situ* loading, synchrotron

## Abstract

Long-term stability of endosseous implants depends on successful bone formation, ingrowth and adaptation to the implant. Specifically, it will define the mechanical properties of the newly formed bone-implant interface. 3D imaging during mechanical loading tests (*in situ* loading) can improve the understanding of the local processes leading to bone damage and failure. In this study, titanium screws were implanted into rat tibiae and were allowed to integrate for 4 weeks with or without the addition of the growth factor Bone Morphogenetic Protein and the bisphosphonate Zoledronic Acid. Samples were subjected to *in situ* pullout using high-resolution synchrotron x-ray tomography at the Tomcat beamline (SLS, PSI, Switzerland) at 30 keV with 25 ms exposure time, resulting in a total acquisition time of 45 s per scan, with a 3.6 μm isotropic voxel size. Using a custom-made loading device positioned inside the beamline, screws were pulled out with 0.05 mm increment, acquiring multiple scans until rupture of the sample. The *in situ* loading protocol was adapted to ensure short imaging time, which enabled multiple samples to be tested with short loading steps, while keeping the total testing time low and reducing dose deposition. Higher trabecular bone content was quantified in the surrounding of the screw in the treated groups, which correlated with increased mechanical strength and stiffness. Differences in screw implantation, such as contact between threads and cortex as well as minor tilt of the screw were also correlated to the mechanical parameters. *In situ* loading enabled the investigation of crack propagation during the pullout, highlighting the mechanical behavior of the interface. Three typical crack types were observed: (1) rupture at the interface of trabecular and cortical bone tissues, close to the screw, (2) large crack inside the cortex connected to the implant, and (3) first failure away from the screw with cracks propagating toward the screw-bone interface. Mechanical properties of *in vivo* integrated bone-metal screws rely on a combination of multiple parameters that are difficult to identify and separate one from the other.

## Introduction

Metallic implants are widely used as surgical treatments, inserted in direct contact with bone to replace deficient joints, to provide support during fracture healing or for dental replacements (Agarwal and García, 2015). Insufficient ingrowth, excessive implant micromotion or infection, amongst other actors, can compromise the long-term implant stability and result in loosening, thereby requiring the implant to be replaced (Mathieu et al., 2014). Strategies to enhance bone integration include using pharmacological treatments, e.g., Bone Morphogenetic Proteins (BMPs). BMPs are anabolic drugs that enhance osteoblast (bone forming cell) activity at the same time as triggering osteoclast (bone resorbing cell) activity that increase bone resorption (Kärrholm et al., 2006). However, the resorption can be controlled by additional antiresorptive drug, such as a bisphosphonate (Harding et al., 2008; Mathavan et al., 2013; Teng et al., 2016). The effect of these drugs are well-characterized in bone chamber, fracture or defect healing models (Harding et al., 2008; Bosemark et al., 2013; Horstmann et al., 2017; Raina et al., 2018), but the effect on bone-implant mechanical stability and resistance is not well-known.

Mechanical properties of the ingrowth and stability of endosseous implants are commonly investigated through *in-vitro* pullout tests, where the primary output is the macroscopic load-displacement response (Iijima et al., 2013; Shea et al., 2014). The challenge is to transform the macroscopic parameters derived from the load-displacement data into local bone-implant mechanical response, which is highly uncertain due to the complex nature of the structure of the bone and the implant-bone interface. Monitoring such loading tests with high-resolution 3D tomographic imaging would help understanding the local processes leading to bone damage and fracture.

X-ray micro-computed tomography (μCT) is the most viable technique to visualize 3D internal structure of trabecular and cortical bone networks (Bouxsein et al., 2010). Synchrotron radiation with a high flux photon beam enables higher resolution and shorter imaging times compared to lab x-ray sources (Peyrin, 2009). Shorter exposure time is of particular interest for *in situ* loading experiments where several repeated scans are carried out since it limits the total duration of a test. This will reduce the amount of drying of the test specimen (thus preserving the mechanical properties) and the sample relaxation during each scan (thus enabling tests that are more comparable to standard tests). Moreover, artifacts observed during x-ray tomography of metallic implants, such as beam hardening, can be reduced using synchrotron radiation, since it provides a monochromatic and parallel beam (Barrett and Keat, 2004; Bernhardt et al., 2004; Dudeck et al., 2014; Nasirudin et al., 2015; Neldam et al., 2015).

Over the past years, synchrotron-based tomographic imaging has been increasingly used to investigate the morphometry of bone tissue (Peyrin et al., 2014; Ma et al., 2016; Giuliani et al., 2018) as well as of the newly formed bone around implants (Bernhardt et al., 2004; Sarve et al., 2011; Dudeck et al., 2014; Neldam et al., 2015). Previously measured parameters are bone-implant contact (BIC) and bone volume fraction (BV/TV) in a region of interest (ROI) up to 1 mm around the implant. The relationship between BIC and/or BV/TV, and mechanical properties is complex and studies show conflicting results (He et al., 2017). Morphometry obtained with imaging alone is thus not sufficient to comprehend the mechanical competence of the interface.

We believe that imaging with concurrent mechanical testing may provide better insight into the deformation processes. Thus, in this study we conducted *in situ* loading of metal implants integrated into rat tibiae using synchrotron based tomographic imaging to investigate the behavior of the bone-implant interface under loading. High-resolution imaging and fast image acquisition were considered key features to keep the observations and mechanical response relevant. Moreover, anabolic and anti-resorptive drug treatments were added to provide a range of local bone formation and mechanical strength.

## Materials and Methods

### Animal Model

Male Sprague Dawley rats (age 8 weeks, average weight 95 ± 19 g, Charles River, Germany) were used in the study. The rats were anesthetized with diazepam and pentobarbitalnatrium. Antibiotic prophylaxis was given as 12.5 mg dihydrostreptomycin and 10 mg procaine benzylpenicillin. Under aseptic conditions, a longitudinal incision was made over the anteromedial aspect of the right proximal tibial metaphysis and a 1.5 mm diameter hole was drilled transversely to the longitudinal axis of the bone, with implantation of a titanium screw (Ø2.6 mm, length 8 mm) (Le Cann et al., 2017).

Animals were divided into three groups: control, BMP and BMP + Za, detailed hereafter. From a larger animal experiment (*n* = 12 per group), a subset of 5, 4 and 6 animals respectively for control, BMP and BMP + Za groups was isolated for this study. Control group received 0.02 mL of NaCl solution inside the drilled hole before screw insertion. BMP group received 50 μg of BMP-7 (Osigraft, Stryker Biotech, Malmö, Sweden) administered in the form of a putty consisting of 2 mg of BMP-7 per 570 mg bovine collagen mixed with 200 mg carboxymethylcellulose (Mathavan et al., 2013). BMP + Za group received the same BMP-7 putty and a Zoledronic Acid systemic injection (0.1 mg/kg) (Zometa, Novartis, Apoteket AB, Sweden) 2 weeks after implantation (Little et al., 2007; Bosemark et al., 2013). The wound was closed, leaving the entire screw subcutaneously. Postoperatively, 4.5 μg buprenorphine was given as analgesic. This research was approved by the Ethics Committee affiliated with the Swedish Board of agriculture (Jordbruksverket, Jönköping, Sweden), under the animal ethics permission number M25-13. All animal care and treatment followed the institutional guidelines. The rats had free access to water and food. The animals were sacrificed after 4 weeks and the tibiae were carefully dissected with the screw in place.

### *In situ* Mechanical Tensile Test Using X-ray Tomography

X-ray tomography was performed at the X02DA TOMCAT beamline, Swiss Light Source (SLS), Paul Scherrer Institut (PSI), Switzerland. One set of imaging parameters was used, namely monochromatic beam energy 30 keV, 100 μm aluminum, 10 μm copper and 10 μm iron beam filters, 3.6 μm isotropic voxel size, 25 ms exposure time. The recorded 1,800 projections covered 180° and resulted in a total acquisition time of 45 s per scan. The field of view (FOV) of the detector was 2.8 × 7.2 mm^2^ (height × width).

Base-line (unloaded) tomographic scans were first conducted on all tibiae (*n* = 15) with the samples in place in the loading device, but before applying any load. Two scans were acquired with a vertical shift to cover the entire screw threads (total FOV 5 mm high).

**Figure 1:**
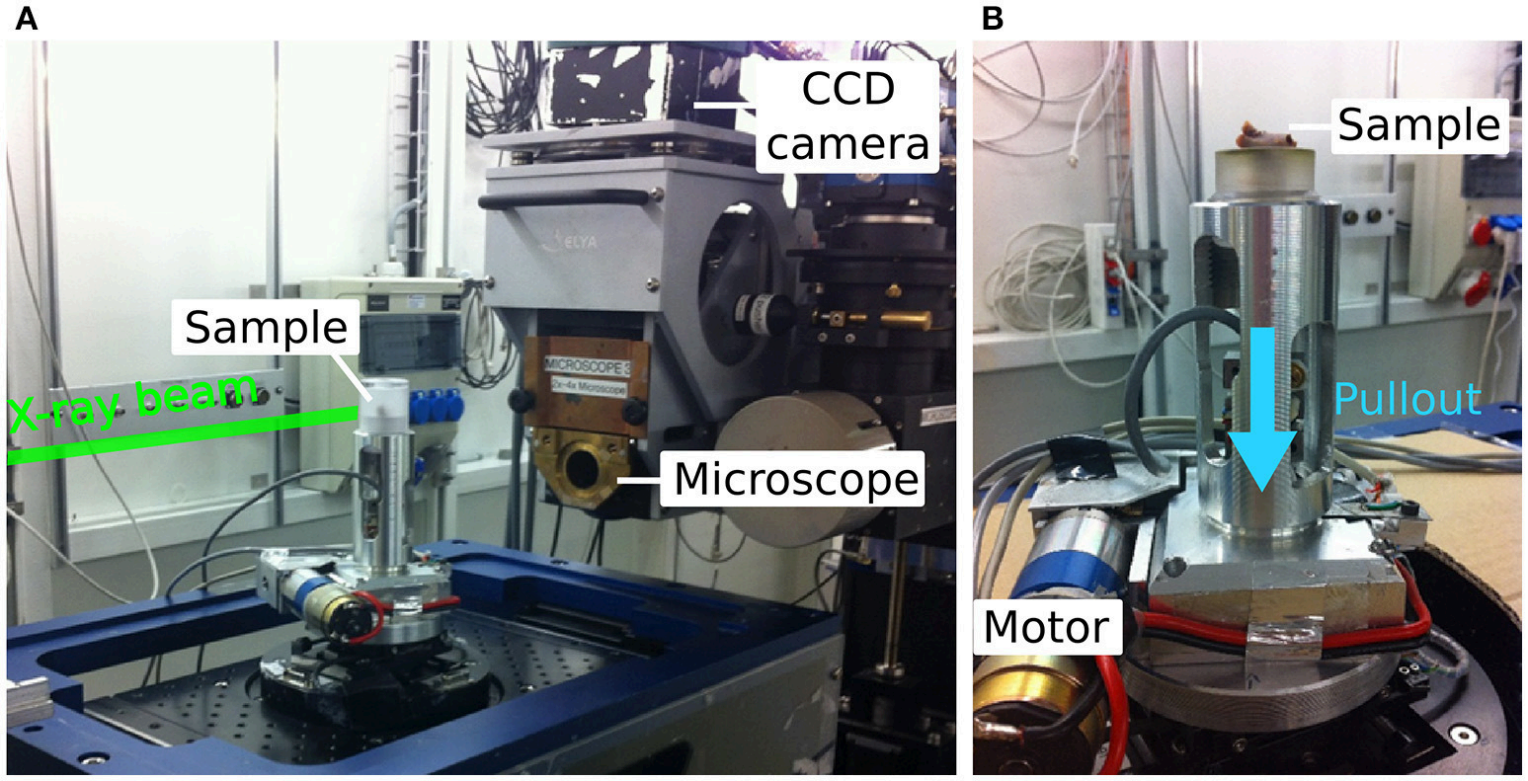
**(A)** Overview of the *in situ* loading set-up at TOMCAT beamline, SLS, PSI, Switzerland. **(B)** Custom made loading device used to pull the screw out of the tibia (sample placement is illustrated without surrounding chamber). The blue arrow indicates the pull-out direction.

Due to limited beamtime, *in situ* screw pullout was conducted on 11 samples from both extreme groups in terms of expected bone formation thus mechanics, the control and BMP + Za groups, using a custom-made loading device (Le Cann et al., 2017) (Figure 1). Each tibia was placed in a cylindrical chamber with the screw head pointing downwards and connected to a hook. A saline steam was inserted in the loading chamber before each test to keep the samples hydrated. Parts of the loading device in the beam path were made of polycarbonate to ensure highest possible X-ray transmission. Samples were centered with the screw threads in the FOV and preloaded to 7 N (6.7 ± 2.8 N) where a first tomographic scan was acquired. Immediately after, a tensile displacement of 0.05 mm was applied to the screw at 0.1 mm/min, followed by another tomographic acquisition. The force and displacement were measured throughout the test using a force transducer and a displacement sensor connected to the hook, and the data were recorded by a custom written LabVIEW program. This step-loading and scanning procedure was repeated until a drop in the force-displacement curve was observed; a final tomography was acquired after failure. The tomographic images were reconstructed using Fourier based regridding algorithm (Marone and Stampanoni, 2012; Marone et al., 2017).

The radiation dose deposited was estimated to be 2 kGy per scan resulting in an estimated maximal total dose per sample of 22 kGy (range 12–38). The estimation was performed for a simplified sample geometry of trabecular bone, consisting of a mixture of adipose tissue (density of 0.9) and bone (density of 1.32) (Lovric et al., 2013).

### Evaluation of Screw Insertion

The unloaded base-line synchrotron scans were rotated to align the screw vertically using an ImageJ plugin (TransformJ v2.1 Erik Meijering, Rotterdam, The Netherlands) and filtered using a 3D median filter with radius 4 pixels (Figure S1 in Supplementary Material). The screw was segmented based on a threshold (Seg3D, SCI Institute's National Institutes of Health/National Institute of General Medical Sciences CIBC Center, Bethesda, MD) and the cortical bone mask was obtained combining manual segmentation every 25 slices subsequently interpolated using MATLAB R2017b (The MathWorks, Inc., Natick, Massachusetts). The contact surface (intersection of the two masks) between the screw threads and the cortex was calculated for every sample.

**Figure 2:**
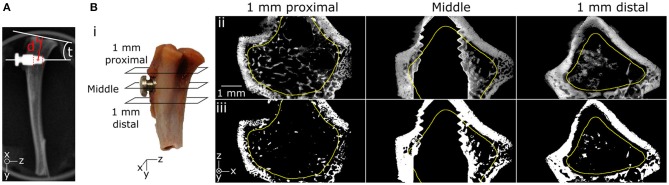
**(A)** Screw insertion parameters showing distance (d) between the screw and the tibial plateau as well as tilt (t) measured on a 2D radiograph. **(B)** Trabecular ROI started 1 mm proximal to the screw edge and ended 1 mm distal to the screw edge (i). ROI included trabecular bone and excluded the cortex as drawn in yellow in original (ii) and segmented (iii), as exemplified in three transversal cuts from the unloaded scan.

Distance between the screw and the tibial plateau as well as initial tilt of the screw after implantation were measured manually on 2D radiographs (pixel size 150 μm, GE Healthcare discovery X-ray) using Fiji (Figure 2A) (Schindelin et al., 2012). For each sample, a tibial plateau line was drawn, passing between the two intercondylar eminences. The middle of the screw threads was defined as the intersection of the axial axis of the screw and the middle of the threaded portion. This point was orthogonally projected onto the tibial plateau line and defined as *d*, the distance between the screw and the tibial plateau. The angle between the axial axis of the screw and the tibial plateau line was defined as *t*, the tilt of the screw.

### Trabecular Bone Content Around the Screw

Bone ingrowth was quantified in all samples using the aligned and median-filtered unloaded base-line scans. A ROI was defined over the screw height, extending 1 mm proximal and distal from the screw, excluding the cortex (Figure 2B). Images were segmented using the same threshold for all samples to remove the background and the screw. Trabecular bone volume fraction (BV/TV) was determined inside the ROIs using the ImageJ plugin BoneJ (Doube et al., 2010).

### Statistics

Non-parametric Mann-Whitney U-tests were used to compare groups. Spearman correlation coefficient tests were used to investigate correlations within the data, using significance level of 0.05 (XLSTAT 2017, Excel, Addinsoft, Paris, France).

## Results

### Analysis of Unloaded Images (*n* = 15)

#### Screw Insertion

**Figure 3:**
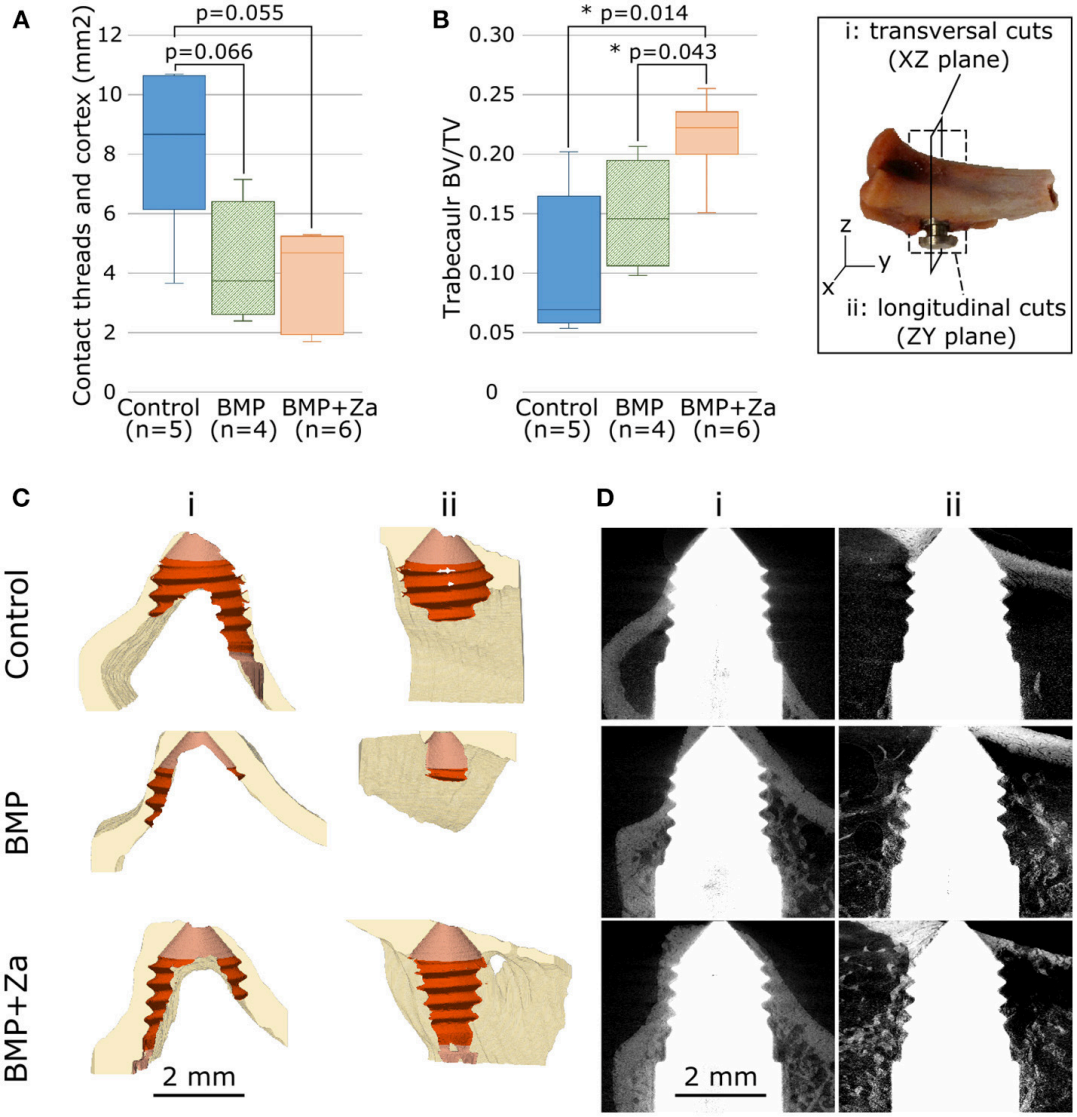
Contact between screw threads and cortex **(A)** and bone volume fraction in ROI defined in Figures 2 **(B)** for each treatment group presented with box-and-whisker plots showing the range of the data, the quartiles, and the median; ^*^represents *p* < 0.05. In insert are illustrated the cutting planes used in **(C,D)**: transversal (i, full line) and longitudinal (ii, dotted line). **(C)** Partial cuts of cortical bone (beige) segmentation to visualize the contact with the screw (light red: whole contact, dark red: with threads) for a typical sample from each treatment group. **(D)** Cuts of raw scans to visualize trabecular bone in the same samples. Data in **(C)** is presented in 3D as (Supplementary Data Sheets S1–S3 for respectively control, BMP and BMP + Za samples).

In the control group, the screws were more deeply inserted, observed visually and confirmed with a higher contact area between threads and cortex (Figures 3A,C). The cortex was visually found to be porous toward the tip of the screw in 3 out of 4 BMP samples and 5 out of 6 BMP + Za samples, but not in any of the control samples. The distance between the screws and the tibial plateau was similar in all groups, with screw implanted 6.5 ± 0.8 mm distally from the plateau (Table 1). The initial tilt of the screw ranged from an insertion almost parallel to the tibial plateau (low tilt, 3.9°) to a maximum of 21° of tilt, with random variation among groups (Table 1).

**Table 1:** Quantification of different screw insertion parameters.

**Treatment group**	**Control (*N* = 5)**	**BMP (*N* = 4)**	**BMP + Za (*N* = 6)**
Distance screw-tibial plateau	6.4 ± 0.7 mm	6.8 ± 0.8 mm	6.5 ± 0.9 mm
Screw tilt compared to tibial plateau	9.6 ± 4.8°	11.2 ± 7.0°	12.9 ± 5.4°

*Data is presented as mean ± SD for each treatment group*.

#### Trabecular Bone Around the Screw

Higher BV/TV was found in the trabecular ROI around the threads in the treated groups compared to control (Figures 3B,D). Image artifacts due to the metal implants were observed in all images, which limited the ability to quantify bone volume in the very close proximity of the screw (Figures 2B, 3D).

### Mechanical Tensile Test Under X-ray Tomographic Imaging (*n* = 11)

**Figure 4:**
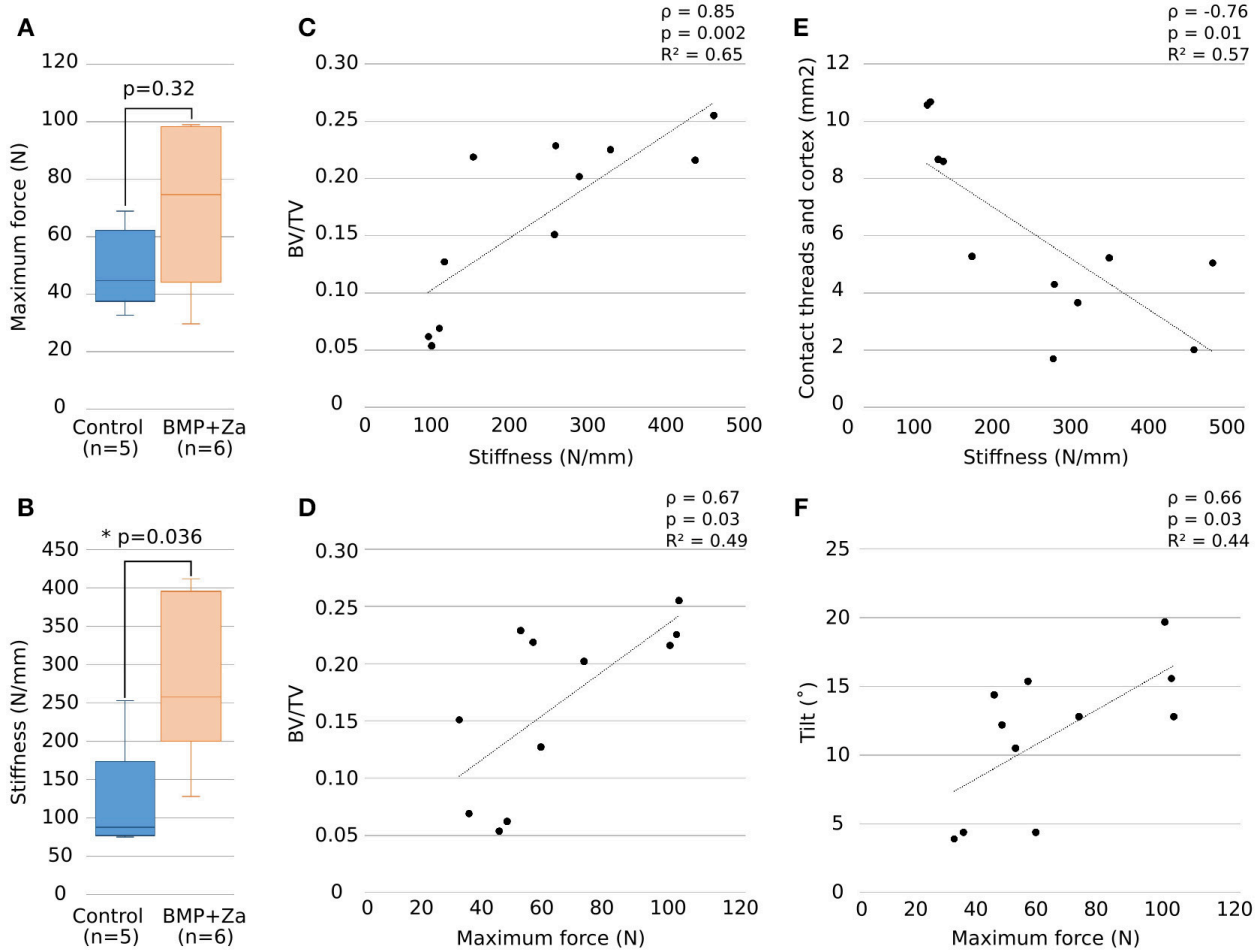
Maximum force and stiffness **(A,B)** presented as box-and-whisker plots showing the range of the data, the quartiles, and the median; ^*^represents *p* < 0.05. Significant (*p* < 0.05) correlations obtained between stiffness **(C,E)** and maximum force **(D,F)** with BV/TV and screw insertion are shown with Spearman correlation coefficient ρ, *p*-value and *R*-squared value of linear regression curve.

For the 11 samples loaded *in situ* (5 controls, 6 BMP + Za), an average of 11 scans (range 6–19) were recorded per sample, leading to an average experimental time of 43 min (range 30–60 min). Stiffness and maximum force were higher in the BMP + Za group compared to controls (*p*-value = 0.036 and *p* = 0.32, respectively, Figures 4A,B) and were positively correlated (*R*^2^ = 0.66, Spearman coefficient 0.81, *p* = 0.002). Moreover, significant correlations were found between the mechanical parameters and inner trabecular BV/TV as well as screw insertion (*p* < 0.05). Specifically, stiffness correlated positively with BV/TV (Figure 4C) and negatively with contact cortex-threads (Figure 4E), maximum force correlated positively with BV/TV (Figure 4D) and tilt (Figure 4F).

**Figure 5:**
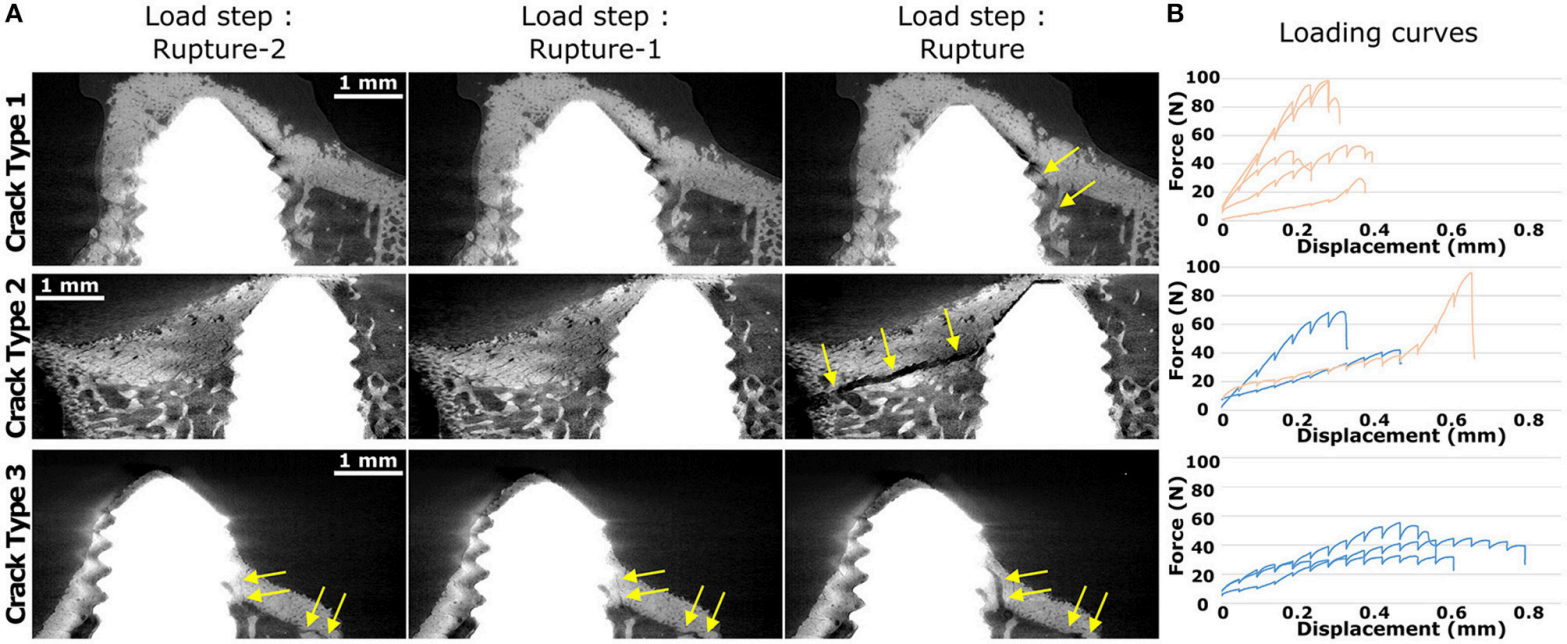
Typical crack patterns from 2D slice cuts **(A)** from two steps before rupture (left) to the step at or immediately after rupture (right). Yellow arrows indicate the main cracks. Data in **(A)** is presented as GIF in Supplementary Data Sheets (S4–S6 for respectively Crack Type 1, Type 2, and Type 3 samples). **(B)** Force vs. displacement curves during *in situ* pullout of all samples in each crack group, where the dark blue curves are control samples and the light orange curves are BMP + Za samples. Crack Type 1 showed rupture close to screw mainly inside trabecular bone. Crack Type 2 failed through large cortical cracks. Crack Type 3 indicates that failure started presumably away from the screw and propagated toward the interface.

Visual inspection of the final scan after failure was used to classify the samples into three groups depending on their crack pattern (Table 2; Figure 5):

- Crack Type 1 (five samples) with rupture at the interface of trabecular and cortical bone and inside the trabecular network close to the screw.
- Crack Type 2 (three samples) presenting rupture from one large cortical crack.
- Crack Type 3 (three samples) where the rupture was presumably initiated away from the screw, with cracks propagating toward the bone-screw interface leading to complete failure.

**Table 2:** Classification of mechanically tested samples by visual inspection of the crack patterns.

**Crack group**			**Crack Type 1**							**Crack Type 2**			**Crack Type 3**		
**Treatment group**			BMP + Za	BMP + Za		BMP + Za		BMP + Za	BMP + Za	NaCl	NaCl	BMP + Za	NaCl	NaCl	NaCl
Cortical cracks		Crack at junction with trabecular bone													
		Small cortical crack													
		Extensive cortical crack													
Trabecular cracks		No cracks visible/little trabecular bone													
		Cracks close to the screw													
		Cracks away from screw													
Appearance of cracks		More than one step before max load													
		One step before max load													
		At max load													

*Crack Type 1: rupture close to screw mainly inside trabecular bone. Crack Type 2: large cortical crack. Crack Type 3: failure away from the screw, propagating toward the interface*.

All samples from Crack Type 1 belonged to the BMP + Za treatment group and these were all samples with a highly porous cortex. Those samples showed no or few cortical cracks compared to other crack groups (Table 2). Samples from Crack Type 2 presented a sudden and large crack in the cortex, not visible before maximal load. All samples from Crack Type 3 were from the control group and exhibited lower BV/TV, higher contact between screw threads and cortex, lower stiffness during pullout as well as higher displacement at maximum force compared to Crack Type 1 (Figure 5B). Furthermore, in Crack Type 3 samples, cracks were observed in the scans already some steps before failure whereas sudden cracks were mostly observed in other crack groups (Figure 5A; Table 2).

## Discussion

In this study the mechanical behavior of the bone-implant interface has been investigated using mechanical testing *in situ* with high-resolution synchrotron-based tomography. Short imaging times enabled short loading step increments and reduced the overall test durations, which enabled the analysis of crack progression during pullout. Analysis of the *in situ* mechanical loading tests on a number of samples with metallic screws integrated into trabecular bone revealed that the overall variations in the mechanical behavior of the construct are dependent on both biological factors (e.g., growth factors) and surgical factors (e.g., screw positioning and insertion). Results confirm that local treatments with BMP and bisphosphonates enhances local bone formation and ingrowth, resulting in improved mechanical properties. Surgical implantation affected the mechanical behavior, which highlights the large variability, uncertainty and complexity of using screw pullout *in vivo* models.

The rat species was chosen to enable easy handling and rapid new bone formation (Wancket, 2015), as well as allow the use of a rather small implant size, which was selected to reduce its X-ray absorption and thereby limiting the artifacts during imaging. The proximal tibial metaphysis in rats consists of cancellous bone with thin cortices, well adapted to study trabecular bone regeneration and ingrowth. The time of ingrowth of 4 weeks was chosen to primarily focus on the bone drugs effect on trabecular bone formation, rather than remodeling. This model has been developed and previously used by our team (Isaksson et al., 2017; Le Cann et al., 2017).

Trabecular bone volume fraction was higher in the treated groups compared to the control group (Figure 3), which agrees well with the literature on the effect of BMP and Za in other animal models (Belfrage et al., 2012; Mathavan et al., 2013; Raina et al., 2018). In addition to increasing osteoblast activity, BMPs also affect the remodeling cycle at large, and thereby trigger an increase in osteoclast activity, which will induce more bone resorption. Remodeling may have initiated already at 4 weeks, and could explain the porous cortex observed at the screw tip in most of the treated samples.

The deep insertion and the small dimensions of the rat bones induced some inevitable contact of the screw threads with the cortical bone. Screws in control samples were found more deeply inserted (Figure 3, Data Sheet S1), which could come from the surgical protocol, where the compressible BMP putty injected after drilling could have restricted the insertion of the screws. Another hypothesis is a potential migration of the screw during the integration, coming from the forces exerted by the muscles on the external part of the screw, which may have been higher in control samples where the amount of trabecular bone available to resist migration was lower.

Both screw insertion parameters and bone volume fraction correlated with the mechanical properties (Figure 4), highlighting their roles on implant mechanical stability. The initial screw tilt was correlated to the maximum force, which is in agreement with a previous study showing that screw insertion angle impacts pullout strength and stiffness (Varghese et al., 2017). According to literature, we were also expecting a positive correlation between the extent of the screw threads-cortex contact and the mechanical properties (Marquezan et al., 2014). However, we observed a negative correlation; samples with higher cortex contact were less stiff (Figure 4E). This is most likely not a cause-and-effect relationship, but rather explained by (1) a primary cortical contact at the tip of the screw in contrast to the beginning of the screw threads, and (2) a negative relationship between cortical contact and trabecular bone volume; samples with high cortical contact were control samples with low trabecular bone volume and vice versa. This underlines the complex nature of implant ingrowth models and the various factors involved. Moreover, trabecular BV/TV positively correlated with mechanical parameters (Figure 4). Combined with a reduced threads-cortex contact (Figure 3) which is in addition porous, and a higher stiffness (Figure 4), these findings support the hypothesis that trabecular bone network has a greater impact on mechanical stability compared to cortical bone, providing higher mechanical support. This agrees well with literature, where the importance of trabecular bone has been highlighted by removing the cortex (Marquezan et al., 2014). However, as trabecular bone ingrowth and screw insertion are intricately linked and both impact the mechanical properties of the bone-implant construct, investigating their separate influence would require the development of different models adapted to only one parameter.

*In situ* loading experiments with x-ray tomography have been used only sparsely to investigate the mechanical properties of the bone-implant interface [e.g., (Du et al., 2015; Joffre et al., 2017)]. Such experiments were conducted on lab-source x-ray tomographs, using lower resolution (usually around 10–25 μm voxel size) and longer imaging time (around 1 h 30 per scan), thus restricting the number of samples analyzed (2–4 per study). The use of synchrotron radiation in this study enables to combine a reduced the imaging time (45 s per scan) with a higher resolution (3.6 μm voxel size), consequently enabling assessment of larger number of samples with an attempt to unravel the effect of the treatments. All 15 samples from control, BMP and BMP + Za treated groups were scanned once unloaded to characterize the microstructure of the newly formed bone and screw insertion parameters, and 11 samples (control and BMP + Za) were loaded *in situ* until rupture. The remaining four samples were not loaded due to limited beamtime. Despite a rather low sample number when comparing to biological studies and studies of pharmacological treatments, this represents to date the largest *in situ* high resolution synchrotron tomographic imaging study done on bone (Nazarian and Müller, 2004; Hussein et al., 2012).

A downside of the high flux synchrotron radiation is that the radiation doses experienced by the tissue are generally much higher than with conventional x-ray sources, with the risk of affecting bone mechanics (Barth et al., 2011; Peña Fernández et al., 2018). We focused on having a short imaging time to limit test duration and radiation to preserve bone mechanics, which led to an average dose deposition of 22 kGy, estimated using a simplified geometry model (cylindrical bone plug). However, this value should be considered as maximum, as the bigger sample sizes and the presence of the metallic implant used in this study reduced the deposited dose substantially. We are thus confident that the dose remained well below the value of 35 kGy (Barth et al., 2011), where no major effect on the mechanical integrity of the bone has been observed (Barth et al., 2011; Peña Fernández et al., 2018).

The samples in this study were visually ranked into three groups according to their crack patterns observed in the tomographic scans during the *in situ* pullout (Figures 5A, Data Sheet S2). The higher porosity of the cortex as well as the less deep insertion of samples from Crack Type 1 could explain the presence of cracks inside the trabecular network rather than the cortex. In a similar but opposite observation, all but one of the remaining samples were control samples, presenting significantly lower trabecular bone and higher contact between the screw threads and cortex, coherent with the observation of large cortical cracks (Table 2). Three samples (Crack Type 3) were assumed to experience the initial cracking away from the interface. This hypothesis is supported by the observation of cracks appearing less suddenly and seen to open in multiple scans close to rupture (Table 2; Figures 5A, Data Sheet S2). Moreover, when looking at the loading curves (Figure 5B), those samples experienced higher displacements at maximum force and longer plastic deformation phases before failure. This could suggest that bone started to fail at a distance away from the screw while the interface was still intact, and that the cracks then propagated toward the screw-bone interface until failure, delaying the rupture of the interface. Some other samples classified in Crack Types 1 and 2 also displayed rather flat loading curves, which could indicate additional failing away from the interface. The rather limited FOV unfortunately did not enable us to confirm this hypothesis. However, we already experienced crack initiation at the tibial plateau in previous experiments using a similar setup but with neutron tomography imaging (Le Cann et al., 2017), which could indicate that these cracks occur due to the experimental setup.

The main limitation of this study is that artifacts were present in the images because of the metallic implant. The high difference in absorption between the metal (highly absorbent) and the bone tissue results in the photon starvation phenomenon (less photons reaching the detector behind the implant), inducing random thin bright and dark streaks in the images (Figure 2B) (Barrett and Keat, 2004). High x-ray energy [e.g., 50–70 keV (Bernhardt et al., 2004; Sarve et al., 2011; Neldam et al., 2015)] is commonly used when scanning metallic components, enabling good transmission through the metal. However, the high energy needs to be combined with a long exposure time to ensure enough contrast, e.g., 4 h scan for a similar size sample in Sarve et al. (2011). This was not compatible with our aim of *in situ* loading experiment while keeping radiation damage to a minimum. Our short exposure time (25 ms/projection, 45 s total scan time) imposed us to use a lower energy (30 keV) to increase contrast in the sample (Boas and Fleischmann, 2012). The artifacts limited the ability to accurately quantify bone ingrowth at the interface and also resulted in an overestimation of the bone content in this region (Bernhardt et al., 2004). Consequently, we extended the ROI for trabecular bone analysis to 1 mm away from the screw (Figures 2B, 3B); since the same implant geometry and imaging parameters were applied to all samples, we assume that the artifacts affect all samples equally. Secondly, the artifacts followed the screw threads during *in situ* loading, which inhibited our ability to use Digital Volume Correlation, as originally planned, to access internal local deformations during loading [e.g., (Roberts et al., 2014; Joffre et al., 2017; Le Cann et al., 2017)]. An extensive range of image processing steps (including mean, median, Gaussian, band pass, edge detection techniques, screw removal in sinograms) were applied to the images to reduce the artifacts, unfortunately with limited success. The use of other imaging techniques less sensitive to metal, such as neutron tomography (Isaksson et al., 2017; Le Cann et al., 2017), as well as other implant materials less sensitive to x-rays are currently investigated to limit artifacts and enable more detailed information of local bone damage mechanisms.

## Conclusions

This study represents the first *in situ* loading of bone and metallic implant interface using synchrotron radiation, and can be used as guidance for future studies. The experimental setup enabled high resolution (pixel size 3.6 μm) with multiple loading steps (step increment 0.05 mm) in a short test time (<60 min per sample). It highlighted that mechanical properties of *in vivo* osseointegrated screws rely on a combination of multiple parameters difficult to apprehend and separate one from the other. It has been shown that not only treatment and bone formation impact the mechanical resistance of the interface, but also the implantation of the screw, such as the contact with cortex, distance from the tibial plateau and angulation of the screw. Screw implantation *in vivo* in small animal models is hardly constant, but such experiments provide advantages including investigation of bone ingrowth and evaluation of treatments on multiple samples. The imaging parameters adapted to repetitive scanning with limited radiation damage induced significant artifacts in the images due to the presence of the metallic implant, which limited the analysis. Therefore, further studies will be conducted to reduce those artifacts and analyze the local mechanical behavior of the bone-implant interface.

## Author Contributions

SLC, ET, SAH, and HI designed the study. MT and OB conducted the animal experiments. SLC, ET, MJT, AP, RM, and HI planned and carried out the *in situ* imaging experiment. SLC analyzed the data and drafted the manuscript. All authors have contributed and agreed to the final manuscript.

### Conflict of Interest Statement

The authors declare that the research was conducted in the absence of any commercial or financial relationships that could be construed as a potential conflict of interest.

## Supplementary Material

Figure S1 Effect of the 3D median filter (*R* = 4 pixels) and the segmentation on one typical slice of a 3D scan.

Data Sheet S1 3D representation of the contact between the cortical bone and the screw for a sample from the control group.

Data Sheet S2 3D representation of the contact between the cortical bone and the screw for a sample from the BMP group.

Data Sheet S3 3D representation of the contact between the cortical bone and the screw for a sample from the BMP + Za group.

Data Sheet S4 Image sequence of one sample cut displaying the 3 last load steps before failure, illustrating the crack appearance and propagation for a sample ranked in Crack Type 1 group, i.e., rupture close to screw, mainly inside trabecular bone.

Data Sheet S5 Image sequence of one sample cut displaying the 3 last load steps before failure, illustrating the crack appearance and propagation for a sample ranked in Crack Type 2 group, i.e., rupture through a large cortical crack.

Data Sheet S6 Image sequence of one sample cut displaying the 4 last load steps before failure, illustrating the crack appearance and propagation for a sample ranked in Crack Type 3 group, i.e., where failure started presumably away from the screw and propagated toward the interface.
