# Supplementary figures and images for: Investigating the Mechanical Characteristics of Bone-Metal Implant Interface Using in situ Synchrotron Tomographic Imaging
Source: Front Bioeng Biotechnol. 2019 Jan 21;6:208. doi: 10.3389/fbioe.2018.00208 (PMC6348316; doi:10.3389/fbioe.2018.00208)

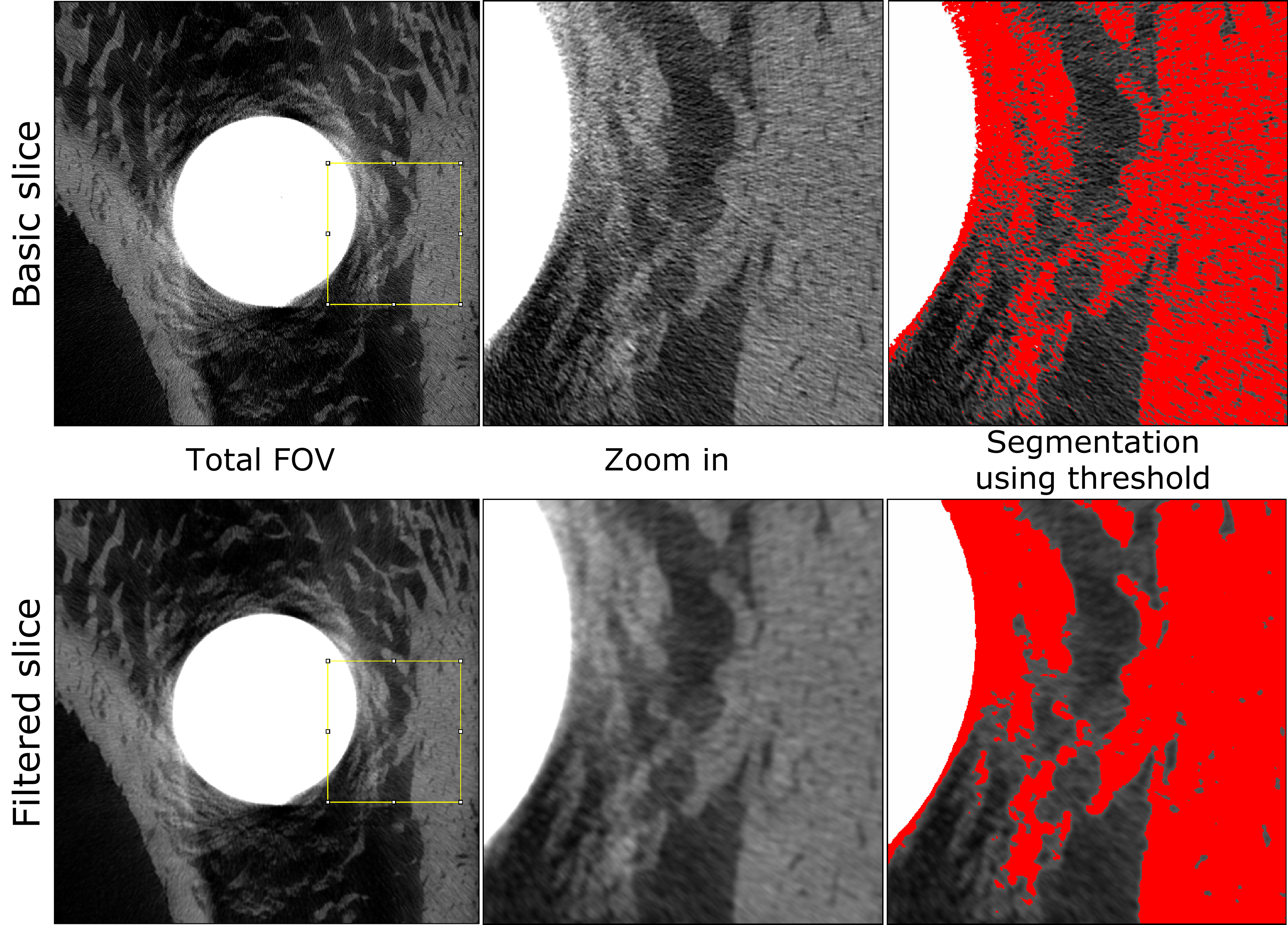

Supplement: Figure S1 — Effect of the 3D median filter (R = 4 pixels) and the segmentation on one typical slice of a 3D scan. [file Image_1.TIF]

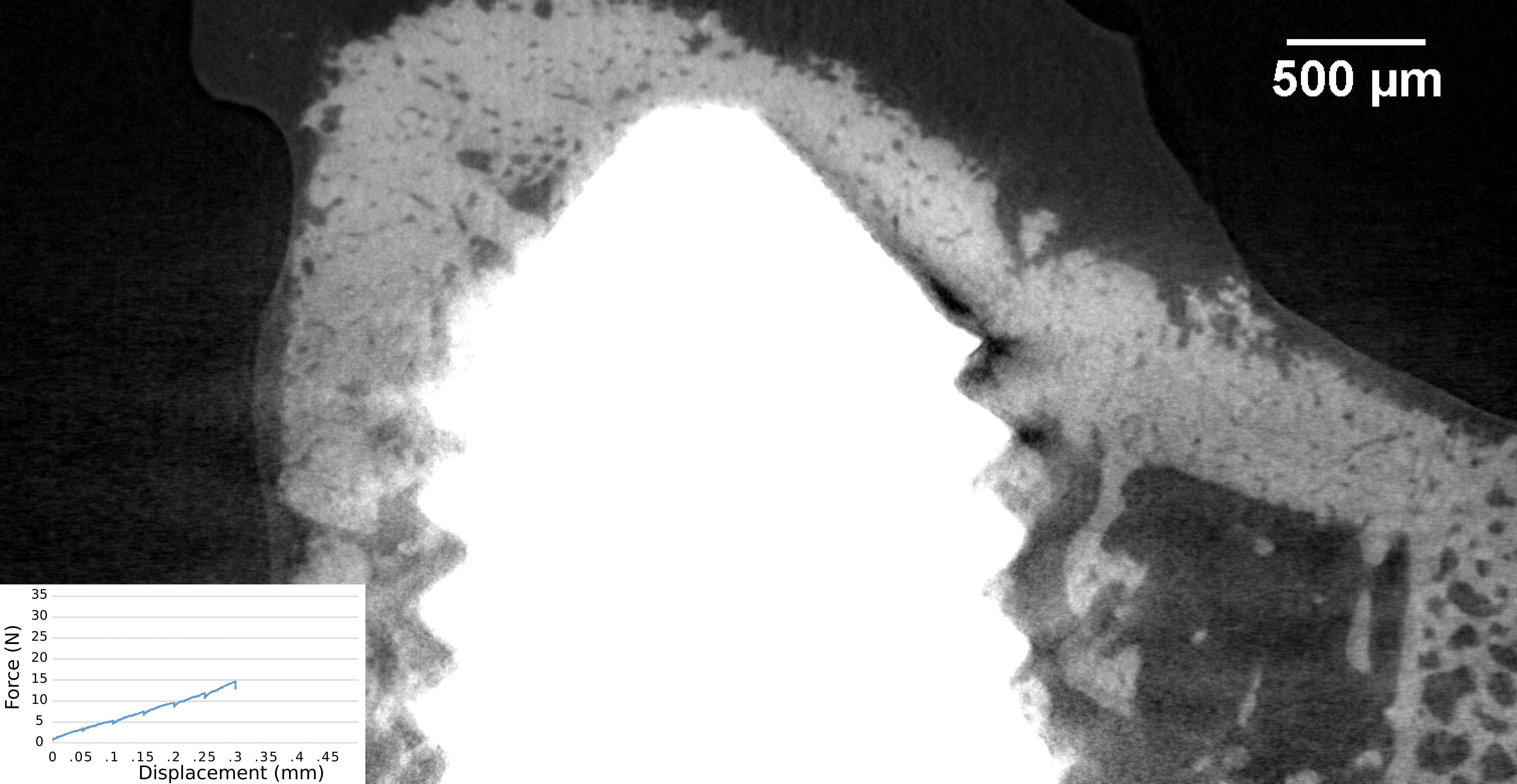

Supplement: Data Sheet S4 — Image sequence of one sample cut displaying the 3 last load steps before failure, illustrating the crack appearance and propagation for a sample ranked in Crack Type 1 group, i.e., rupture close to screw, mainly inside trabecular bone. [file Data_Sheet_4.ZIP › S4_CrackType1_gif.gif]

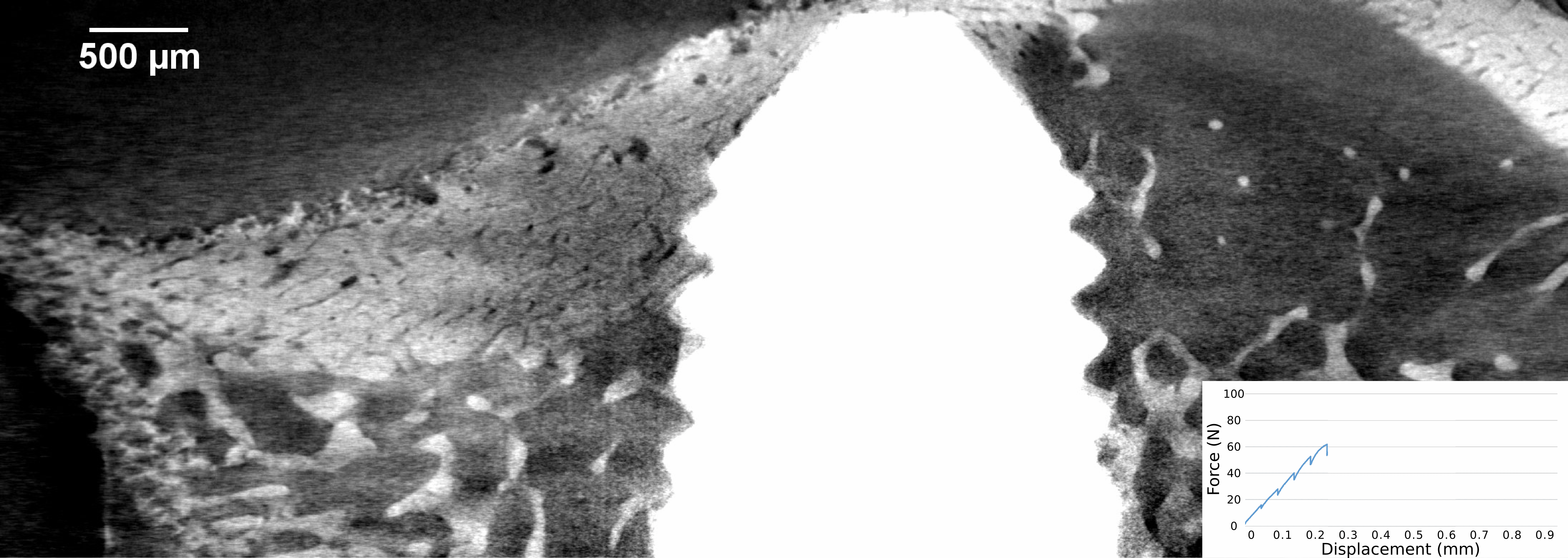

Supplement: Data Sheet S5 — Image sequence of one sample cut displaying the 3 last load steps before failure, illustrating the crack appearance and propagation for a sample ranked in Crack Type 2 group, i.e., rupture through a large cortical crack. [file Data_Sheet_5.ZIP › S5_CrackType2_gif.gif]

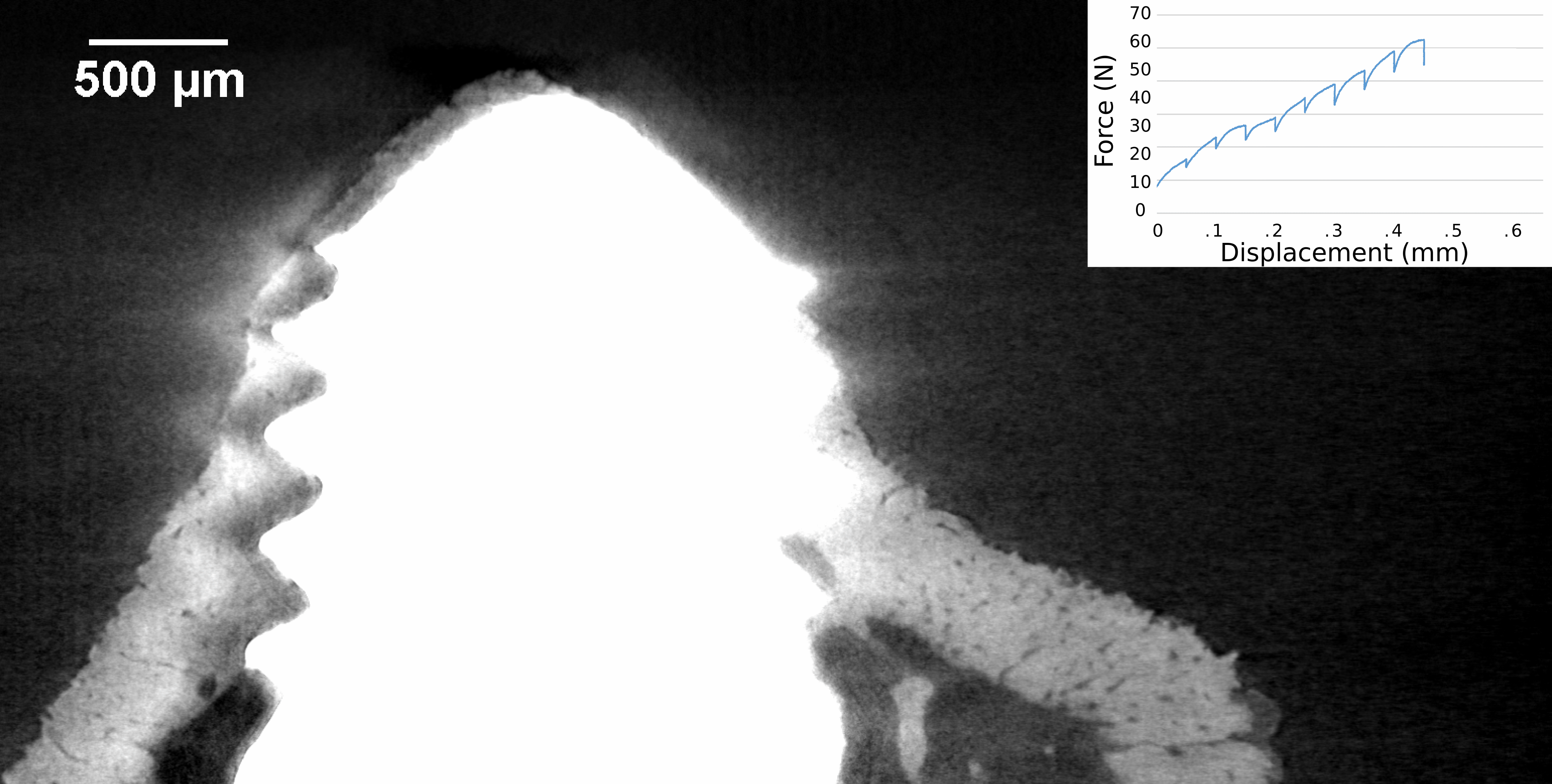

Supplement: Data Sheet S6 — Image sequence of one sample cut displaying the 4 last load steps before failure, illustrating the crack appearance and propagation for a sample ranked in Crack Type 3 group, i.e., where failure started presumably away from the screw and propagated toward the interface. [file Data_Sheet_6.ZIP › S6_CrackType3_gif.gif]
